# Does weather matter? Exploring associations between season and meteorological factors and psoriatic arthritis disease activity

**DOI:** 10.1136/rmdopen-2026-006967

**Published:** 2026-10-23

**Authors:** Batoul Hojeij, Jolanda J Luime, Reinhard Bos, Lindy-Anne Korswagen, Marijn Vis, Jessica Bijsterbosch, Paul Baudoin, Jos H Van Der Kaap, Petra Kok, Yvonne Goekoop-Ruiterman, Marc R Kok, Ilja Tchetverikov

**Affiliations:** 1 Department of Rheumatology, Erasmus Medical Center, Rotterdam, The Netherlands; 2 Department of Rheumatology, Frisius Medical Center, Leewarden, The Netherlands; 3 Department of Rheumatology, Franciscus Gasthuis and Vlietland, Rotterdam, The Netherlands; 4 Department of Rheumatology, Amphia Hospital, Breda, The Netherlands; 5 Department of Rheumatology, Reumazorg Zuid West Nederland, Goes, The Netherlands; 6 Department of Rheumatology, Admiraal De Ruyter Hospital, Goes, The Netherlands; 7 Department of Rheumatology, Reinier de Graaf Hospital, Delft, The Netherlands; 8 Department of Rheumatology, Haga Hospital, The Hague, The Netherlands; 9 Department of Rheumatology, Maasstad Hospital, Rotterdam, The Netherlands; 10 Department of Rheumatology, Albert Schweitzer Hospital, Dordrecht, The Netherlands

**Keywords:** Psoriatic Arthritis, Epidemiology, Pain, Arthritis

## Abstract

**Objective:**

To investigate the associations between season and psoriatic arthritis (PsA) disease activity, and whether certain meteorological factors are associated with these seasonal differences. Secondary objectives include assessing associations between season and mood.

**Methods:**

A retrospective longitudinal study was conducted including 935 patients with PsA from the Dutch South West Psoriatic Arthritis cohort from 2013 to 2023. Disease activity was assessed using Psoriatic Arthritis Disease Activity Score (PASDAS) and Disease Activity in Psoriatic Arthritis (DAPSA) at diagnosis (baseline) and over 3 years of follow-up. Season was assessed during the month of each visit, and meteorological factors (temperature, relative humidity, wind speed, atmospheric pressure, sunshine duration) were assessed during the day of and week and month before the visit, using data from the Royal Netherlands Meteorological Institute. Mood was assessed using the Hospital Anxiety and Depression Scale.

**Results:**

At diagnosis, there was no significant difference in PsA incidence and disease activity (PASDAS and DAPSA) between seasons. During follow-up, the multivariable analyses showed higher DAPSA in winter (β 2.59; 95% CI 0.74 to 4.43), spring (β 1.95; 95% CI 0.001 to 3.90) and autumn (β 1.93; 95% CI 0.17 to 3.69) compared with summer. Analyses of DAPSA subcomponents showed significantly higher Visual Analog Scale (VAS) patient pain and global assessment in autumn and higher swollen joint count 66 and C-reactive protein in winter compared with summer. Season was not significantly associated with PASDAS, nor with mood. Meteorological factors were not significantly associated with PASDAS and DAPSA.

**Conclusion:**

Season was associated with PsA disease activity, being lower in summer. Meteorological factors were not associated with PsA disease activity in our study sample.

**WHAT IS ALREADY KNOWN ON THIS TOPIC**

- Previous studies suggest that weather may impact rheumatic symptoms, but the evidence for psoriatic arthritis (PsA) is limited and inconclusive.

**WHAT THIS STUDY ADDS**

- PsA disease activity was lower in summer compared with other seasons.
- Temperature, humidity, wind speed, atmospheric pressure and sunshine duration were not significantly associated with PsA disease activity.

**HOW THIS STUDY MIGHT AFFECT RESEARCH, PRACTICE OR POLICY**

- This study provides context for considering seasonal differences in PsA disease activity in research and practice.
- Further studies in different geographic regions and with more frequent measurements are warranted to confirm the findings.

## Introduction

 Psoriatic arthritis (PsA) is a chronic immune-mediated inflammatory disease associated with psoriasis.^1^ The disease activity of PsA can vary over time. Factors such as obesity, arthritis therapy and gut microbiome have been shown to contribute to PsA disease activity.^2–4^ In clinical practice, patients with PsA also complain that changes in weather conditions influence their symptoms.^5^ However, literature on the effects of weather on PsA is limited and inconclusive.^6 7^

Weather may influence PsA disease activity through its effects on physical measures such as swollen and tender joints or patient-reported outcomes (PROs) such as pain.^5 8–11^ However, the mechanisms by which weather influences disease activity are poorly understood. One pathway is through the sensory receptors.^12^ For example, environmental temperature and atmospheric pressure are detected by thermosensitive ion channels and mechanoreceptors in the skin.^12–15^ These sensory receptors can impact nociceptive signalling, influencing pain sensitivity and thereby contributing to changes in disease activity.^8 12–15^ Weather conditions such as season variations and cold have been also shown to influence inflammatory biomarker levels.^12 16–18^ Weather can also influence factors such as mood, physical activity and sleep which have been shown to influence disease activity.^19–26^ For example, the probability of having a bad mood has been negatively associated with increased temperature.^19^ Light physical activity has been positively associated with temperature, sunshine and cloud cover and negatively with wind speed.^20^ Moreover, physical activity is higher in summer compared with other seasons.^21^ Season has been also associated with sleep patterns, showing longer sleep duration in winter.^22 23^

Literature on the effect of weather on arthritis symptoms is ambiguous.^9–11^ Cumulative evidence suggests an association between weather and osteoarthritis pain,^11^ whereas findings are inconsistent for rheumatoid arthritis (RA) pain.^9 27^ Studies on other arthritis symptoms such as tender and swollen joints, stiffness, as well as overall disease activity score are limited.^9^ Ambiguity in findings on weather may be due to variations in the range of weather conditions across the geographic regions studied, and in how weather is measured. For example, weather was assessed by season, and/or specific meteorological factors such as minimum, maximum or average values, on a daily or weekly basis, or based on changes over time.^6 19 28 29^

In PsA, two studies assessed the difference in disease activity between summer and winter; one of these studies also assessed the impact of meteorological factors within each season.^6 7^ The findings were inconsistent in terms of season, and the impact of meteorological factors was not clinically significant.^6 7^ In the present study, we investigated the associations between the four seasons and PsA disease activity, and examined whether certain meteorological factors (during the day of, week and month prior to the visit) are associated with these differences. As a secondary objective, we assessed the associations between season and mood of patients with PsA.

## Methods

### Population and setting

We conducted a retrospective longitudinal study using data of 938 patients newly diagnosed with PsA from the multicentre Dutch South West Psoriatic Arthritis (DEPAR) cohort, between 2013 and 2023. The DEPAR cohort is an ongoing prospective cohort study that collects real-world data on disease activity and long-term outcomes in patients with early PsA.^30^ Patients were recruited from centres in the southwest region of the Netherlands. Inclusion criteria were PsA diagnosis according to the treating physician (without applying classification criteria for psoriatic arthritis (CASPAR) criteria), at least 18 years of age, not receiving disease-modifying antirheumatic drugs (DMARDs) for joint symptoms at inclusion and having sufficient command of the Dutch language. Patients with no data on the season and meteorological factors because of missing visit date at all study visits were excluded (n=3).

### Weather

Season was classified according to the meteorological criteria in the Northern hemisphere into winter (December–February), spring (March–May), summer (June–August) and autumn (September–November).^31^ The season during the month of each visit was assessed. Data on meteorological factors were obtained from the Royal Netherlands Meteorological Institute (KNMI) using weather stations located near the residential postal addresses of patients. The nearest weather station was determined by selecting the closest station from the KNMI stations registry to the city corresponding to the patient residential postal address. When a KNMI weather station was located within the patient’s city, that station was assigned directly. The meteorological factors included: temperature (°C), relative humidity (%), wind speed (m/s), atmospheric pressure adjusted for sea level (hPa) and sunshine duration (hours).^32^ Meteorological factors were assessed by calculating the average exposure for the day of, and for the week and month before each visit.

### Disease activity measures

PsA disease activity outcomes were assessed at diagnosis (baseline visit), and at the end of the first, second and third year of follow-up (online supplemental table 1). Disease activity was measured using PsA-specific composite scores: Disease Activity in Psoriatic Arthritis (DAPSA) and Psoriatic Arthritis Disease Activity Score (PASDAS).^8^ We also assessed the individual components of these scores: patient-reported Visual Analog Scale (VAS) pain, VAS patient global, VAS physician global, number of swollen (swollen joint count 66 (SJC66)) and tender joints (tender joint count 68 (TJC68)), and C-reactive protein (CRP) levels, short form-36 (SF-36) physical component scale (PCS), Leeds Enthesitis Index (LEI), and Leeds dactylitis Index (LDI).^8^

### Mood

Mood was assessed using three selected questions from the Hospital Anxiety and Depression Scale at the end of the first, second and third year of follow-up, to examine if the associations between season and disease activity can be (partly) attributable to seasonal effects on mood.^33^ We chose questions that reflect general mood state, without focusing solely on depression or anxiety. The questions included: ‘I feel tense or wound up’, ‘I feel cheerful’ and ‘I can sit at ease and feel relaxed’.

### Statistical analysis

Descriptive statistics were used to analyse the baseline characteristics of the study population.

Linear regression models were used to analyse the associations between weather and PsA disease activity at diagnosis (ie, the baseline visit). Multivariable models were adjusted for age, sex, education years, geographic origin (Dutch/Non-Dutch), body mass index (BMI), smoking (yes/no) and alcohol consumption (yes/no).

Crude and multivariable linear mixed-effect models were used to assess associations between weather and PsA disease activity at the first, second and third year follow-up visits. The models included all available disease activity measurements obtained during follow-up visits as the dependent variable. Participants were included if at least one disease activity measurement was available during follow-up. The participant ID and follow-up visit were included as random effects in these models. Crude models were adjusted for baseline disease activity and follow-up visit. Multivariable models were additionally adjusted for age, sex, education years, geographic origin (Dutch/Non-Dutch), BMI, smoking (yes/no), and alcohol consumption (yes/no), and arthritis therapy at previous visit (methotrexate, biologics, glucocorticoids, conventional synthetic DMARDs, no DMARD use).

To assess whether the findings were robust to underlying distribution, we performed sensitivity analyses for the multivariable models that showed statistically significant associations. The models were re-estimated using heteroscedasticity-consistent (robust) standard errors and generalised linear mixed models (GLMMs) with a Tweedie distribution.

Follow-up visits with available disease activity measurements but missing data for one or more covariates were included in the analyses, and missing covariate values were handled using multiple imputations (20 imputation sets with 20 iterations each). The imputations were performed using Multivariate Imputation by Chained Equations (MICE) with predictive mean matching. Missing follow-up visits, disease activity measurements and meteorological factors (online supplemental table 2) were not included in the analyses.

All analyses were conducted in STATA V.18.5. Multiple imputations and GLMMs were performed in R V.4.4.2.

## Results

### Baseline characteristics

Table 1 shows the characteristics of patients at diagnosis. A total of 935 patients with PsA who had at least one disease activity measurement and study visit that could be assigned to a season were included. The median age of the patients was 51 years, and 50% were women. Patients were more likely of Dutch origin (82%, n=762), and to be diagnosed with oligoarthritis (n=323, 35%). The median BMI was in the overweight range (28 kg/m^2^). The median SJC66 was 2 and TJC68 was 3, and the median CRP level was 4 mg/L. 53% of patients did not use DMARDs at diagnosis, and 41% were using methotrexate. 258 (28%) patients were diagnosed during autumn, 240 (26%) were diagnosed during winter, 223 (24%) were diagnosed during spring and 213 (22%) were diagnosed during summer (p value=0.477). There was no difference in the characteristics of patients at diagnosis between seasons (p values>0.05).

**Table 1:** Baseline characteristics of the study population at diagnosis

	Total (n=935)	Autumn (n=258)	Winter (n=240)	Spring (n=223)	Summer (n=213)	*P value
Age (years), median (IQR)	51 (39, 60)	51 (40, 61)	51 (40, 61)	51 (39, 59)	49 (37, 60)	0.629
Missing, n (%)	1 (0.13)					
Sex (women), n (%)	472 (50)	142 (55)	122 (51)	107 (48)	101 (47)	0.319
Missing, n (%)	1 (0.11)					
Education (years), median (IQR)	12 (10, 15)	13 (10, 16)	12 (10, 15)	13 (11, 16)	12 (11, 15)	0.307
Missing, n (%)	123 (13)	28 (11)	31 (13)	39 (17)	25 (12)	
Geographic origin (Dutch), n (%)	762 (82)	217 (84)	198 (83)	172 (77)	174 (82)	0.889
Missing, n (%)	124 (13)	28 (11)	31 (13)	39 (17)	26 (12)	
Tender Joint count 68, median (IQR)	3 (1, 7)	3 (1, 7)	4 (2, 8)	3 (1, 8)	3 (1, 8)	0.364
Missing, n (%)	7 (1)	2 (1)	2 (1)	3 (1)		
Swollen joint count 66, median (IQR)	2 (1, 5)	2 (1, 5)	2 (1, 5)	2 (1, 4)	2 (1, 5)	0.547
Missing, n (%)	7 (1)	2 (1)	2 (1)	3 (1)		
CRP (mg/L)	4 (0.4, 11)	5 (0, 12)	4 (1, 10)	4 (0, 10)	4 (1, 12)	0.732
Missing, n (%)	113 (12)	29 (11)	31 (13)	29 (13)	23 (11)	
Diagnosis, n (%)						0.099
Monarthritis	161 (17)	44 (17)	34 (14)	42 (19)	41 (19)	
Oligoarthritis	323 (35)	91 (35)	92 (39)	76 (34)	64 (30)	
Polyarthritis	237 (25)	63 (24)	68 (28)	50 (22)	56 (26)	
Enthesitis	70 (7)	27 (11)	14 (6)	13 (6)	16 (8)	
Dactylitis	97 (10)	26 (10)	22 (9)	22 (10)	6 (3)	
Axial	27 (3)	3 (1)	5 (2)	13 (6)	27 (13)	
Missing	20 (2)	4 (2)	5 (2)	7 (3)	3 (1)	
BMI, median (IQR)	28 (25, 32)	28 (25, 31)	27 (25, 31)	27 (25, 32)	28 (25, 32)	0.969
Missing, n (%)	10 (1)	1 (0.4)	2 (1)	7 (3)		
Smoking (yes), n (%)	176 (19)	57 (22)	36 (15)	40 (18)	43 (20)	0.272
Missing, n (%)	123 (13)	28 (11)	31 (13)	39 (17)	25 (12)	
Alcohol consumption (yes), n (%)	669 (72)	186 (72)	170 (71)	151 (68)	161 (76)	0.311
Missing, n (%)	123 (13)	28 (11)	31 (13)	39 (17)	25 (12)	
Medication, n (%)						
Methotrexate, n (%)	379 (41)	101 (39)	106 (44)	95 (43)	77 (36)	0.307
csDMARD	38 (4)	11 (4)	12 (5)	7 (3)	8 (4)	0.776
Biologics	23 (2)	9 (3)	4 (2)	6 (3)	4 (2)	0.549
Glucocorticoids	62 (7)	23 (9)	17 (7)	14 (6)	8 (4)	0.162
No DMARDs	497 (53)	137 (53)	119 (50)	115 (52)	126 (59)	0.209
Missing, n(%)	1 (0.11)					

* Between seasons.

BMI, body mass index; CRP, C-reactive protein; csDMARD, conventional synthetic disease-modifying antirheumatic drug; DMARDs, disease-modifying antirheumatic drugs.

### Season and PsA

Figure 1 shows the difference in DAPSA and PASDAS scores between the seasons. At diagnoses, 720 of the 935 participants had DAPSA and/or PASDAS measurement. Season was not significantly associated with PASDAS and DAPSA in the crude and multivariable analyses at diagnosis (table 2). During follow-up, 621 of the 935 participants contributed a total of 1272 visits with a DAPSA and/or PASDAS measurement, comprising 391 visits (n=226 participants) in autumn, 330 (n=191) in winter, 257 (n=151) in spring and 294 (n=158) in summer. The median follow-up duration of participants from diagnosis was 2.98 years, with 37% (n=232) having three follow-up visits, 30% (n=187) having two follow-up visits and 33% (n=202) having one follow-up visit. In the multivariable analyses, DAPSA was higher in winter (β 2.59; 95% CI 0.74 to 4.43), spring (β 1.95; 95% CI 0.001 to 3.90) and autumn (β 1.93; 95% CI 0.17 to 3.69) compared with summer (table 3). Season was not associated with PASDAS in the crude and multivariable analyses (table 3).

**Figure 1:**
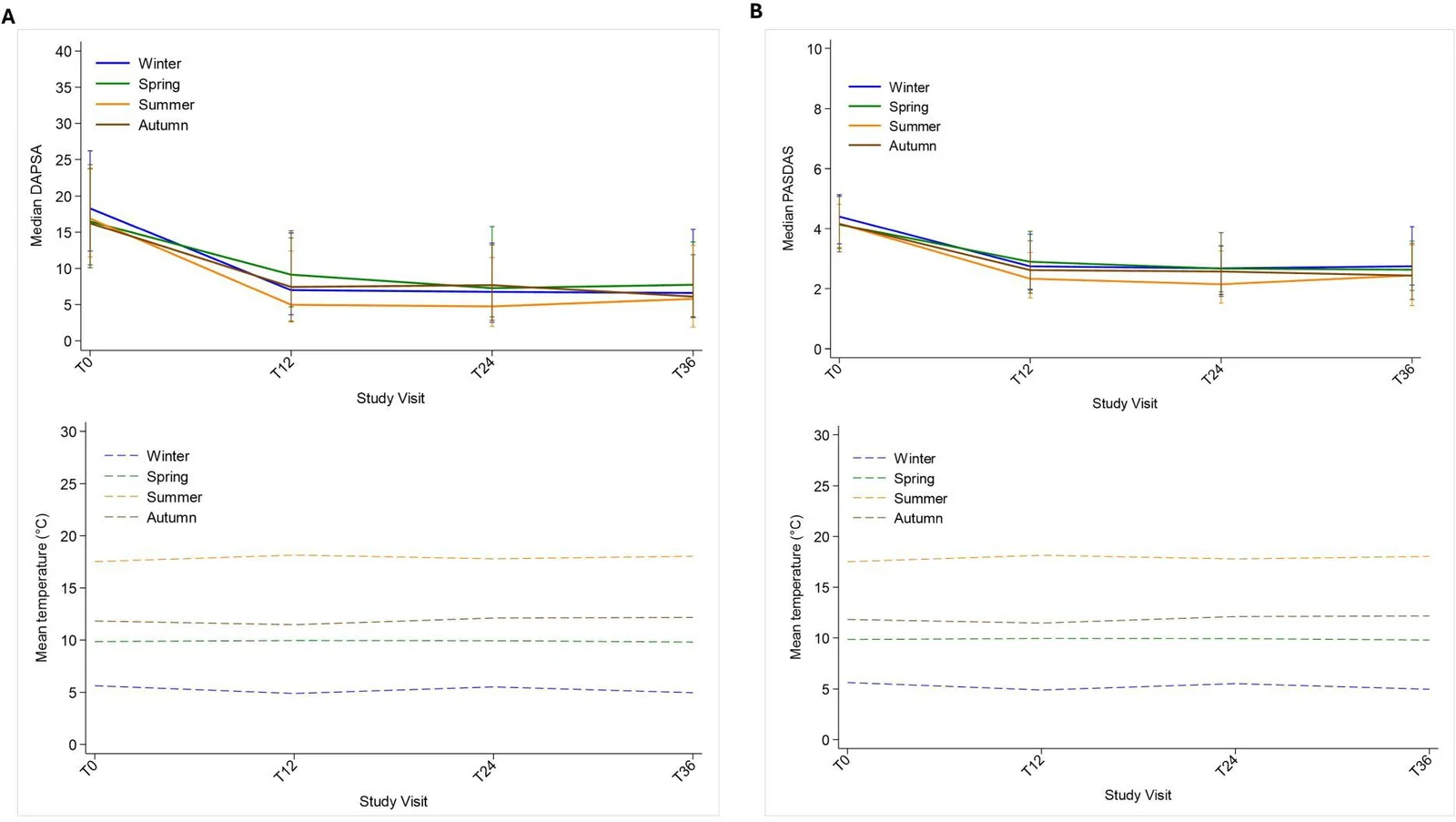
Difference in DAPSA (A) and PASDAS (B) scores between the four seasons. DAPSA, Disease Activity index for Psoriatic Arthritis; PASDAS, Psoriatic Arthritis Disease Activity.

**Table 2:** Associations between season and psoriatic arthritis disease activity scores at diagnosis

Disease activity	Autumn	Winter	Spring	Summer
Crude model				
DAPSA				
Participants, n	200	174	165	175
β (95% CI)	0.06 (−2.43 to 2.56)	2.09 (−0.49 to 4.67)	−0.24 (−2.85 to 2.38)	Ref
PASDAS				
Participants, n	182	162	147	169
β (95% CI)	0.03 (−0.22 to 0.28)	0.20 (−0.05 to 0.46)	0.08 (−0.18 to 0.35)	Ref
Adjusted model				
DAPSA				
Participants, n	200	174	165	175
β (95% CI)	−0.24 (−2.70 to 2.23)	2.06 (−0.48 to 4.61)	−0.24 (−2.81 to 2.33)	Ref
PASDAS				
Participants, n	182	162	147	169
β (95% CI)	0.003 (−0.24 to 0.25)	0.20 (−0.05 to 0.45)	0.08 (−0.17 to 0.34)	Ref

Adjusted model: age, sex, education years, geographic origin, body mass index, smoking and alcohol consumption.

DAPSA, Disease Activity index for Psoriatic Arthritis; PASDAS, Psoriatic Arthritis Disease Activity Score.

**Table 3:** Associations between season and psoriatic arthritis disease activity scores during the 3-year follow-up period

Disease activity	Autumn	Winter	Spring	Summer
Crude model				
DAPSA				
Visits/participants, n	377/165	323/146	248/97	283/125
β (95% CI)	1.61 (−0.15 to 3.36)	2.59 (0.74 to 4.45)**	2.50 (0.56 to 4.43)*	Ref
PASDAS				
Visits/participants, n	373/162	319/139	243/96	285/119
β (95% CI)	0.10 (−0.10 to 0.30)	0.21 (−0.008 to 0.42)	0.17 (−0.05 to 0.39)	Ref
Adjusted model				
DAPSA				
Visits/participants, n	377/165	323/146	248/97	283/125
β (95% CI)	1.93 (0.17 to 3.69)*	2.59 (0.74 to 4.43)**	1.95 (0.001 to 3.90)*	Ref
PASDAS				
Visits/participants, n	373/162	319/139	243/96	285/119
β (95% CI)	0.16 (−0.04 to 0.35)	0.20 (−0.005 to 0.41)	0.10 (−0.12 to 0.32)	Ref

Crude model: baseline disease activity and study visit.

Adjusted model: baseline disease activity, study visit, age, sex, education years, geographic origin, body mass index, smoking, alcohol consumption and arthritis therapy.

Bold values indicate statistical significance (*p value<0.05, **p value<0.01).

DAPSA, Disease Activity index for Psoriatic Arthritis; PASDAS, Psoriatic Arthritis Disease Activity Score.

Further analyses into the subcomponents of DAPSA showed that, in autumn VAS pain (β 6.36; 95% CI 2.55 to 10.17) and global (β 4.66; 95% CI 1.05 to 8.27) were higher, whereas the SJC66 (β 0.78; 95% CI 0.10 to 1.47) and CRP (β 1.40; 95% CI 0.12 to 2.68) were higher in winter, compared with summer (table 4). Season was not associated with the additional components from PASDAS (ie, VAS physician, LEI, LDI and SF36-PCS) in the multivariable analyses (online supplemental table 3).

**Table 4:** Associations between season and DAPSA components during the 3-year follow-up period

Disease activity	Autumn	Winter	Spring	Summer
Crude model				
VAS patient pain				
Visits/participants, n	443/182	383/169	305/119	329/143
β (95% CI)	5.72 (1.87 to 9.56)**	4.48 (0.35 to 8.61)*	4.12 (−0.11 to 8.34)	Ref
VAS patient global				
Visits/participants, n	460/191	396/175	319/127	344/146
β (95% CI)	4.06 (0.46 to 7.66)*	2.40 (−1.46 to 6.27)	3.23 (−0.74 to 7.20)	Ref
SJC66				
Visits/participants, n	511/211	438/186	386/154	396/164
β (95% CI)	0.16 (−0.48 to 0.79)	0.79 (0.12 to 1.44)*	0.54 (−0.14 to 1.21)	Ref
TJC68				
Visits/participants, n	511/211	438/186	386/154	396/164
β (95% CI)	0.43 (−0.48 to 1.34)	0.68 (−0.28 to 1.64)	0.69 (−0.29 to 1.67)	Ref
CRP				
Visits/participants, n	441/191	372/161	316/122	339/143
β (95% CI)	0.58 (−0.53 to 1.70)	1.14 (−0.03 to 2.31)	0.87 (−0.34 to 2.07)	Ref
Adjusted model				
VAS patient pain				
Visits/participants, n	443/182	383/169	305/119	329/143
β (95% CI)	6.36 (2.55 to 10.17)**	3.65 (−0.42 to 7.72)	3.16 (−1.04 to 7.38)	Ref
VAS patient global				
Visits/participants, n	460/191	396/175	319/127	344/146
β (95% CI)	4.66 (1.05 to 8.27)*	2.05 (−1.79 to 5.89)	1.98 (−2.01 to 5.97)	Ref
SJC66				
Visits/participants, n	511/211	438/186	386/154	396/164
β (95% CI)	0.30 (−0.36 to 0.95)	0.78 (0.10 to 1.47)*	0.55 (−0.16 to 1.25)	Ref
TJC68				
Visits/participants, n	511/211	438/186	386/154	396/164
β (95% CI)	0.67 (−0.25 to 1.59)	0.73 (−0.23 to 1.70)	0.78 (−0.21 to 1.78)	Ref
CRP				
Visits/participants, n	441/191	372/161	316/122	339/143
β (95% CI)	0.55 (−0.68 to 1.77)	1.40 (0.12 to 2.68)*	0.75 (−0.59 to 2.09)	Ref

Crude model: baseline component measure and study visit.

Adjusted model: baseline component measure, study visit, age, sex, education years, geographic origin, body mass index, smoking, alcohol consumption and arthritis therapy.

Bold values indicate statistical significance (*p value<0.05, **p value<0.01).

CRP, C-reactive protein; DAPSA, Disease Activity index for Psoriatic Arthritis; SJC, swollen joint count; TJC, tender joint count; VAS, Visual Analog Scale.

### Meteorological factors and PsA

Online supplemental tables 4–7 present the associations between individual meteorological factors and PsA disease activity. The average temperature, relative humidity, atmospheric pressure, sunshine duration and wind speed on the day of the visit and the weekly and monthly averages prior to the visit, were not significantly associated with PASDAS, DAPSA or individual DAPSA components (VAS patient pain and global, SJC66, TJC68 and CRP) in the crude and multivariable analyses at diagnosis and during follow-up (p values>0.05). An exception was a statistically significant association between wind speed on the average month prior to visit and CRP in the crude (β 0.51; 95% CI 0.11 to 0.91) and multivariable analyses (β 0.41; 95% CI 0.01 to 0.82) during follow-up.

### Season and mood

No significant association was observed between season and mood assessment questions (online supplemental table 8).

## Discussion

This real-world cohort study of patients with PsA provides evidence that seasonal variation may influence disease activity. We observed significantly lower DAPSA score, VAS patient pain and global assessments, SJC66 and CRP levels during summer. These results were close to clinical relevance.^34 35^ Meteorological factors (temperature, relative humidity, wind speed, atmospheric pressure and sunshine duration) on the day of, week and month before visit were not associated with PsA disease activity. Moreover, seasonal variations were not associated with mood.

Number of studies in patients with rheumatic diseases have observed effect of season on disease activity and PROs (eg, DAS28, SJC, TJC, gout flares, pain).^36–39^ In PsA, only two studies assessed the associations between season and disease activity, both conducted in Canada, with inconsistent findings: one reported lower disease activity in winter compared with summer, while the other one found no difference.^6 7^ In contrast, our results showed lower disease activity in summer compared with other seasons. This difference in disease activity measures may partly explain these discrepancies. For example, we observed significant associations between season and DAPSA, but not PASDAS. This is likely explained by the PASDAS components: enthesitis, dactylitis and SF-36 physical component, which showed no seasonal variation. Consistent with our findings, Joly-Chevrier *et al* showed no differences in patient pain or physician global assessment between summer and winter.^6^ Notably, we found associations between season and patient global VAS, but not physician global VAS, consistent with findings by Iikune *et al* in patients with RA.^36^ The reason behind this observation is unclear, but it highlights the discordance between patient and physician perceptions of seasonal effects on disease activity.

The associations between season and disease activity were observed during follow-up under established disease management, but not at diagnosis where patients have uncontrolled disease activity and treatment has not yet been initiated. Consequently, differences in disease activity are more likely to reflect the severity and heterogeneity of the underlying disease at presentation, which seem to have greater influence than seasonality. Besides, individual meteorological factors showed no significant associations with disease activity, although we found associations between season and PsA disease activity during follow-up. It could be that PsA disease activity is influenced by discrete differences in weather such as those that occur between seasons or extreme weather conditions, rather than gradual variation in individual meteorological factors.^15^ Also, season captures the combined influence of multiple meteorological factors which together may have a stronger impact compared with any single factor.^9 40^ We also assessed whether seasonal effects on disease activity could be explained by the effect of season on mood, and found no association. This suggests that other factors such as lifestyle, seasonal flues or air pollution may be implicated in this association. For example, physical activity and alcohol consumption have been associated with reduced rheumatic disease activity, and their levels are higher during summer,^21 24 41 42^ whereas winter influenza vaccination can lead to short-term increase in rheumatic disease activity.^43 44^It is worth noting that the higher CRP levels observed during winter may be due to influenza rather than joint inflammation. These higher CRP levels may partly explain the higher disease activity observed during winter. Nevertheless, we found that the swollen joint count was also higher during winter, which attributes to the higher PsA disease activity. Air pollution also varies seasonally and may impact rheumatic disease activity.^45 46^

One study in PsA found no clinically significant effects of temperature or humidity on PROs or disease activity in summer or winter.^6^ Studies in RA showed inconsistent results between meteorological factors and RA symptoms (eg, joint and swelling, stiffness).^9^ A recent meta-analysis found no significant effect of temperature and relative humidity on RA pain,^27^ whereas another one in osteoarthritis found a significant association with osteoarthritis pain.^11^ The differences in weather effects on different arthritis types can be partly explained by their unique pathophysiological mechanisms.

The mechanism underlying the association between season and arthritis symptoms requires further investigation. Oliviero *et al* found increased cytophagocytic mononuclear cells in synovial fluid of patients with rheumatic diseases during spring and autumn,^17^ while Wyse *et al* observed higher CRP and neutrophil levels in winter compared with summer, with lymphocytes peaking in spring and being lowest in autumn.^18^ Besides, Dopico *et al* showed widespread seasonal immune gene expression changes of peripheral blood mononuclear cells, with proinflammatory markers predominating in winter compared with summer.^47^ Pain, commonly used as a surrogate marker of disease activity, has been the primary focus of fundamental and epidemiological studies on weather effects. In a rat model of knee arthritis, Kim *et al* showed that exposure to ambient cold temperature increased synovial proinflammatory cytokines and activated spinal microglia, promoting inflammatory pain.^16^ Environmental factors such as temperature and humidity activate thermoreceptors and mechanoreceptors, triggering the sympathetic nervous system that affect pain sensitivity and immune function.^12 47^ Cold weather also induces peripheral vasoconstriction, reducing blood flow (to reduce heat loss) and aggravating neuropathic pain.^48–50^ Seasonal variation in vitamin D levels (25(OH)D) may further influence the symptoms; Calton *et al* showed that the increase in 25(OH)D levels between summer and winter have been associated with reduced levels of pro-inflammatory markers (eg, interleukin (IL)-6, IL-10, monocyte chemoattractant protein-1 (MCP-1)).^51^ The decline in 25(OH)D levels during winter is possibly due to reduced daylight exposure and outdoor activities in winter; however, we found no association sunshine duration and PsA disease activity. Possible explanations relate to characteristics of the weather in the Netherlands, including cloud cover even during the summer months which reduces ultraviolet B (UVB) exposure despite longer daylight months, as well as cooler summer days that lead to wearing more covering clothing.

This study investigated the weather hypothesis in PsA using a large sample of patients and PsA-specific disease activity measures. Weather was assessed through seasonal variations and meteorological factors over multiple time intervals. Moreover, because this is a retrospective study, disease activity was collected before weather data were analysed, reducing the likelihood that patient reports were influenced by weather and minimising reporting bias. Limitations of this study include assessing weather in a single geographic region with a temperate climate, which limits the generalisability to regions with different climates. Meteorological data were obtained from nearby weather stations based on the patient residential postal address, without accounting for indoor environments, or time spent away from residence (eg, work, travel). The levels of 25(OH) D levels were also not seasonally measured in this study. Moreover, the majority of participants had their follow-up visits within 1-year window (±2 months) (online supplemental table 1), and therefore were assessed during the same season across follow-up visits. Consequently, this limited the ability to assess intraindividual seasonal variation and intraindividual short-term symptom fluctuations. Nevertheless, we may speculate that multiple factors influenced by season are shared across the population (eg, holiday periods, daylight exposure, infections, mood) and would therefore be expected to vary within individuals across seasons. Finally, the analysis did not account for the presence of osteoarthritis which is common in older patients. Osteoarthritis-related pain is known to be sensitive to weather conditions, with temperature, humidity and barometric pressure being shown to influence symptom severity.^11^ Consequently, the observed seasonal differences in pain-related outcomes may be partially driven by osteoarthritis symptoms rather than PsA disease activity. Nevertheless, subgroup analyses by age showed that the associations between season and DAPSA remained significant only during autumn among patients younger than 45 years (online supplemental table 9). Besides, we did not consider the use of non-arthritis medications (eg, NSAIDs, antidepressants, sleep medication) in our analyses which may also impact PROs. For example, higher disease activity may result in greater pain, depressive symptoms and sleep difficulties, leading to increased medication use, while these medications may in turn alleviate these PROs.

Future research with different geographic regions and more frequent follow-ups is warranted. Indeed, meteorological factors can change from day to day and may impact disease activity accordingly. Using digital monitoring tools (eg, unobtrusive digital biomarkers, smartphone-based questionnaires) provides a promising approach for remote and temporal assessment of disease activity across diverse regions. Further research on seasonal effects is also recommended to examine mediating factors such as lifestyle and infections which can vary across seasons and subsequently influence disease activity. Besides, since the associations between season and disease activity were close to clinical significance and the literature in this area is limited, further studies are required to determine whether an observational approach is adequate or whether treatment adjustment should be considered across seasons.

In conclusion, this study using real-world data suggests that PsA disease activity varies by season, being lower in summer. Despite these seasonal variations, meteorological factors (temperature, relative humidity, wind speed, atmospheric pressure and sunshine duration) did not show significant associations with PsA disease activity in our study sample. In addition, mood did not vary by season. Evidence on the associations between season and PsA disease activity contributes to understanding the environmental factors influencing PsA outcomes and can assist in better disease management. It is recommended that healthcare providers recognise seasonal fluctuations in PsA disease activity, particularly during winter and autumn when it seems to be higher.

## Supplementary material

10.1136/rmdopen-2026-006967 online supplemental file 1

## Data Availability

Data are available upon reasonable request.
