# Supplementary material for: Does weather matter? Exploring associations between season and meteorological factors and psoriatic arthritis disease activity
Source: RMD Open. 2026 Oct 23;12(4):e006967. doi: 10.1136/rmdopen-2026-006967 (PMC13711580; doi:10.1136/rmdopen-2026-006967)
Supplement: Supplementary file 1 [file rmdopen-12-4-s001.pdf]

Supplementary Table 1. Time interval between follow-up visits

Visit	n	Days Mean (SD)	Follow-up within one-year window (10-14 months) n(%)
Baseline-Year 1	537	370 (16)	528 (98%)
Year 1-Year 2	414	366 (19)	372 (89%)
Year 2-Year 3	321	366 (17)	283 (88%)

Supplementary Table 2. Overview of available and missing meteorological days for weekly and monthly calculations

	Temperature	Relative humidity	Wind speed	Atmospheric pressure	Sunshine duration
Weekly average (7-day window), n(%)					
Baseline					
Complete 7 days	457 (63%)	457 (63%)	484 (67%)	456 (63%)	408 (57%)
Missing (0 days available)	263 (37%)	263 (37%)	236 (33%)	264 (37%)	312 (43%)
Follow-up					
Complete 7 days	926 (73%)	926 (73%)	979 (77%)	924 (73%)	849 (67%)
Missing (0 days available)	346 (27%)	346 (27%)	293 (23%)	348 (27%)	423 (33%)
Monthly Average (30-day window), n(%)					
Baseline					
Complete 30 days	456 (63%)	456 (63%)	483 (67%)	455 (63%)	407 (56.9%)
28 days	1 (0.1%)	1 (0.1%)	1 (0.1%)	1 (0.1%)	1 (0.1%)
Missing (0 days available)	263 (36.9%)	263 (36.9%)	236 (32.9%)	264 (36.9%)	312 (43%)
Follow-up					
Complete 30 days	926 (73%)	926 (73%)	979 (77%)	924 (73%)	847 (66.8%)
29 days	0	0	0	0	2 (0.2%)
Missing (0 days available)	346 (27%)	346 (27%)	293 (23%)	348 (27%)	423 (33%)

Supplementary Table 3. Associations between season and other PASDAS components during the three years follow-up period

Disease activity	Autumn	Winter	Spring	Summer
Crude model				
VAS physician global				
Visits/participants, n	499/204	431/179	363/145	388/160
β (95%CI)	-1.00 (-2.85, 0.84)	1.20 (-0.75, 3.16)	0.07 (-1.91, 2.06)	Ref
Enthesitis				
Visits/participants, n	511/210	437/185	374/147	393/164
β (95%CI)	0.0002 (-0.15, 0.16)	-0.06 (-0.22, 0.10)	0.004 (-0.16, 0.17)	Ref
Dactylitis				
Visits/participants, n	511/209	438/186	373/147	394/163
β (95%CI)	0.006 (-0.007, 0.02)	-0.0008 (-0.02, 0.01)	-0.0007 (-0.02, 0.01)	Ref
SF-36 physical component				
Visits/participants, n	461/187	392/170	326/126	356/146
β (95%CI)	-0.23 (-1.41, 0.96)	0.10 (-1.20, 1.40)	-0.47 (-1.79, 0.84)	Ref
Adjusted model				
VAS physician global				
Visits/participants, n	204 (499)	179 (431)	145 (363)	160 (388)
β (95%CI)	-0.62 (-2.40, 1.17)	0.56 (-1.33, 2.44)	-0.17 (-2.11, 1.76)	Ref
Enthesitis				
Visits/participants, n	210 (511)	185 (437)	147 (374)	164 (393)
β (95%CI)	-0.01 (-0.17, 0.14)	-0.09 (-0.26, 0.08)	-0.07 (-0.24, 0.10)	Ref
Dactylitis				
Visits/participants, n	511/209	438/186	373/147	394/163
β (95%CI)	0.01 (-0.00004, 0.02)	0.007 (-0.004, 0.02)	0.008 (-0.004, 0.02)	Ref
SF-36 physical component				
Visits/participants, n	461/187	392/170	326/126	356/146
β (95%CI)	-0.64 (-1.83, 0.55)	-0.05 (-1.34, 1.24)	-0.18 (-1.50, 1.14)	Ref

Crude model: baseline component measure and study visit.

Adjusted model: baseline component measure, study visit, age, sex, education years, geographic origin, BMI, smoking, and alcohol consumption, and arthritis therapy.

Supplementary Table 4. Associations between meteorological factors and psoriatic arthritis disease activity scores at diagnosis

Disease activity	Temperature	Relative humidity	Wind speed	Atmospheric pressure	Sunshine duration
Crude model					
Day of visit					
PASDAS					
Participants, n	420	420	445	420	373
β (95%CI)	0.005 (-0.01, 0.02)	0.003 (-0.009, 0.01)	-0.02 (-0.07, 0.03)	0.0003 (-0.01, 0.01)	0.02 (-0.01, 0.05)
DAPSA					
Participants, n	453	453	480	452	404
β (95%CI)	0.13 (-0.07, 0.33)	0.06 (-0.07, 0.18)	-0.24 (-0.77, 0.29)	0.07 (-0.05, 0.19)	0.19 (-0.12, 0.49)
Week prior to visit					
PASDAS					
Participants, n	420	420	445	420	373
β (95%CI)	0.005 (-0.02, 0.03)	0.005 (-0.01, 0.02)	0.02 (-0.04, 0.09)	-0.00001 (-0.02, 0.02)	-0.03 (-0.07, 0.02)
DAPSA					
Participants, n	453	453	480	452	404
β (95%CI)	0.11 (-0.10, 0.33)	-0.0004 (-0.17, 0.17)	0.50 (-0.9, 1.20)	-0.03 (-0.19, 0.13)	-0.28 (-0.74, 0.18)
Month prior to visit					
PASDAS					
Participants, n	420	420	445	420	373
β (95%CI)	0.01 (-0.01, 0.03)	-0.001 (-0.02, 0.02)	0.01 (-0.08, 0.11)	-0.01 (-0.04, 0.01)	-0.03 (-0.08, 0.02)
DAPSA					
Participants, n	453	453	480	452	404
β (95%CI)	0.13 (-0.09, 0.36)	-0.03 (-0.22, 0.16)	0.32 (-0.62, 1.26)	-0.17 (-0.45, 0.11)	-0.29 (-0.81, 0.24)
Adjusted model					
Day of visit					
PASDAS					
Participants, n	420	420	445	420	373
β (95%CI)	0.007 (-0.01, 0.03)	0.0001 (-0.01, 0.01)	-0.01 (-0.07, 0.03)	-0.004 (-0.01, 0.01)	0.03 (-0.004, 0.05)
DAPSA					
Participants, n	453	453	480	452	404
β (95%CI)	0.13 (-0.06, 0.33)	0.04 (-0.09, 0.16)	-0.26 (-0.78, 0.26)	0.06 (-0.07, 0.18)	0.25 (-0.05, 0.55)

Week prior to visit					
PASDAS					
Participants, n	420	420	445	420	373
β (95%CI)	0.007 (-0.01, 0.03)	0.002 (-0.01, 0.02)	0.02 (-0.04, 0.09)	-0.005 (-0.02, 0.01)	-0.03 (-0.07, 0.02)
DAPSA					
Participants, n	453	453	480	452	404
β (95%CI)	0.11 (-0.10, 0.33)	-0.02 (-0.19, 0.14)	0.50 (-0.18, 1.18)	-0.09 (-0.25, 0.07)	-0.28 (-0.73, 0.17)
Month prior to visit					
PASDAS					
Participants, n	420	420	445	420	373
β (95%CI)	0.01 (-0.01, 0.03)	-0.005 (-0.02, 0.01)	0.01 (-0.07, 0.10)	-0.02 (-0.05, 0.01)	-0.02 (-0.07, 0.03)
DAPSA					
Participants, n	453	453	480	452	404
β (95%CI)	0.14 (-0.08, 0.36)	-0.06 (-0.25, 0.13)	0.35 (-0.56, 1.27)	-0.24 (-0.51, 0.04)	-0.24 (-0.76, 0.28)

Adjusted model: age, sex, education years, geographic origin, BMI, smoking, and alcohol consumption.

Supplementary Table 5. Associations between meteorological factors on the day of visit, and PASDAS and DAPSA and it components during the three years follow-up period

Disease activity	Temperature	Relative humidity	Wind speed	Atmospheric pressure	Sunshine duration
Crude model					
PASDAS					
Visits/participants, n	365/897	365/897	388/948	364/896	332/820
β (95%CI)	-0.01 (-0.02, 0.005)	0.002 (-0.01, 0.01)	0.01 (-0.02, 0.04)	-0.001 (-0.01, 0.004)	0.004 (-0.01, 0.02)
DAPSA					
Visits/participants, n	901/371	901/371	954/395	370/899	825/339
β (95%CI)	-0.06 (-0.16, 0.03)	0.001 (-0.05, 0.05)	0.10 (-0.13, 0.33)	-0.02 (-0.07, 0.02)	-0.03 (-0.15, 0.10)
VAS pain					
Visits/participants, n	1056/422	1056/422	1127/450	421/1054	960/384
β (95%CI)	0.07 (-0.20, 0.33)	-0.04 (-0.17, 0.10)	-0.43 (-1.00, 0.13)	-0.05 (-0.17, 0.07)	0.04 (-0.28, 0.36)
VAS global					
Visits/participants, n	1093/441	1093/441	1165/469	440/1091	995/401
β (95%CI)	-0.06 (-0.31, 0.19)	0.05 (-0.08, 0.19)	-0.51 (-1.06, 0.05)	-0.01 (-0.13, 0.10)	0.001 (-0.31, 0.31)
SJC-66					
Visits/participants, n	1245/492	1245/492	1321/521	491/1242	1136/448
β (95%CI)	-0.007 (-0.05, 0.03)	-0.01 (-0.03, 0.01)	0.001 (-0.09, 0.09)	-0.002 (-0.02, 0.02)	-0.007 (-0.06, 0.05)
TJC-68					
Visits/participants, n	1245/492	1245/492	1321/521	491/1242	1136/448
β (95%CI)	-0.01 (-0.07, 0.04)	-0.0006 (-0.03, 0.03)	0.005 (-0.13, 0.14)	-0.01 (-0.04, 0.02)	-0.02 (-0.09, 0.06)
CRP					
Visits/participants, n	1061/426	1061/426	1117/451	425/1058	972/389
β (95%CI)	-0.06 (-0.14, 0.02)	0.0006 (-0.05, 0.05)	-0.06 (-0.28, 0.15)	0.03 (-0.01, 0.08)	0.006 (-0.11, 0.12)
Adjusted model					
PASDAS					
Visits/participants, n	365/897	365/897	388/948	364/896	332/820
β (95%CI)	-0.01 (-0.02, 0.005)	0.003 (-0.004, 0.01)	0.005 (-0.03, 0.04)	-0.001 (-0.01, 0.005)	0.0004 (-0.02, 0.02)
DAPSA					
Visits/participants, n	371 (901)	371 (901)	395 (954)	370/899	339 (825)
β (95%CI)	-0.06 (-0.16, 0.03)	0.003 (-0.05, 0.06)	0.13 (-0.11, 0.36)	-0.02 (-0.07, 0.02)	-0.03 (-0.16, 0.09)

VAS pain					
Visits/participants, n	422/1056	422/1056	450/1127	421/1054	384/960
β (95%CI)	0.11 (-0.15, 0.38)	-0.05 (-0.19, 0.08)	-0.43 (-0.99, 0.14)	-0.05 (-0.17, 0.07)	0.07 (-0.26, 0.40)
VAS global					
Visits/participants, n	441 /1093	441 /1093	469 /1165	440/1091	401 /995
β (95%CI)	-0.03 (-0.28, 0.22)	0.04 (-0.09, 0.17)	-0.48 (-1.04, 0.08)	-0.02 (-0.13, 0.10)	0.06 (-0.25, 0.38)
SJC-66					
Visits/participants, n	492 /1245	492 /1245	521 /1321	491/1242	448 /1136
β (95%CI)	-0.007 (-0.04, 0.03)	-0.01 (-0.03, 0.008)	0.02 (-0.06, 0.10)	-0.007 (-0.02, 0.01)	-0.007 (-0.06, 0.04)
TJC-68					
Visits/participants, n	492 /1245	492 /1245	521 /1321	491/1242	448 /1136
β (95%CI)	-0.02 (-0.08, 0.03)	0.004 (-0.03, 0.03)	0.03 (-0.10, 0.17)	-0.02 (-0.04, 0.01)	-0.005 (-0.08, 0.07)
CRP					
Visits/participants, n	426 /1061	426 /1061	451 /1117	425/1058	389 /972
β (95%CI)	-0.07 (-0.16, 0.02)	-0.002 (-0.06, 0.05)	-0.01 (-0.24, 0.22)	0.04 (-0.01, 0.08)	-0.007 (-0.14, 0.12)

Crude model: baseline PASDAS or DAPSA/component measure and study visit.
Adjusted model: baseline PASDAS or DAPSA/component measure, study visit, age, sex, education years, geographic origin, BMI, smoking, and alcohol consumption, and arthritis therapy.

Supplementary Table 6. Associations between meteorological factors on the average week before visit, and PASDAS and DAPSA and it components during the three years follow-up period.

Disease activity	Temperature	Relative humidity	Wind speed	Atmospheric pressure	Sunshine duration
Crude model					
PASDAS					
Visits/participants, n	365/897	365/897	388/948	364/896	332/820
β (95%CI)	-0.01 (-0.03, 0.003)	-0.001 (-0.01, 0.01)	0.03 (-0.01, 0.08)	0.001 (-0.01, 0.01)	-0.002 (-0.03, 0.02)
DAPSA					
Visits/participants, n	901/371	901/371	954/395	370/899	825/339
β (95%CI)	-0.10 (-0.21, 0.005)	-0.01 (-0.08, 0.06)	0.34 (-0.007, 0.68)	-0.01 (-0.07, 0.05)	-0.10 (-0.30, 0.10)
VAS pain					
Visits/participants, n	1056/422	1056/422	1127/450	421/1054	960/384
β (95%CI)	0.009(-0.28, 0.30)	-0.03 (-0.21, 0.16)	0.005 (-0.86, 0.87)	-0.10 (-0.25, 0.06)	-0.24 (-0.75, 0.28)
VAS global					
Visits/participants, n	1093/441	1093/441	1165/469	440/1091	995/401
β (95%CI)	-0.04 (-0.31, 0.23)	0.02 (-0.16, 0.20)	0.07 (-0.77, 0.90)	-0.06 (-0.21, 0.10)	-0.06 (-0.55, 0.43)
SJC-66					
Visits/participants, n	1245/492	1245/492	1321/521	491/1242	1136/448
β (95%CI)	-0.02 (-0.06, 0.02)	-0.01 (-0.04, 0.01)	0.03 (-0.11, 0.16)	-0.002 (-0.03, 0.02)	0.01 (-0.07, 0.09)
TJC-68					
Visits/participants, n	1245/492	1245/492	1321/521	491/1242	1136/448
β (95%CI)	-0.009 (-0.07, 0.05)	-0.01 (-0.06, 0.03)	0.06 (-0.14, 0.26)	0.005 (-0.03, 0.04)	0.02 (-0.09, 0.14)
CRP					
Visits/participants, n	1061/426	1061/426	1117/451	425/1058	972/389
β (95%CI)	-0.05 (-0.14, 0.04)	-0.001 (-0.07, 0.06)	0.23 (-0.07, 0.53)	0.02 (-0.04, 0.08)	-0.05 (-0.22, 0.12)
Adjusted model					
PASDAS					
Visits/participants, n	365/897	365/897	388/948	364/896	332/820
β (95%CI)	-0.01 (-0.03, 0.002)	0.004 (-0.01, 0.01)	0.02 (-0.02, 0.07)	0.001 (-0.01, 0.01)	-0.01 (-0.03, 0.02)
DAPSA					
Visits/participants, n	901/371	901/371	954/395	370/899	825/339
β (95%CI)	-0.10 (-0.20, 0.002)	0.02 (-0.05, 0.09)	0.29 (-0.05, 0.63)	-0.02 (-0.08, 0.04)	-0.13 (-0.32, 0.06)

VAS pain					
Visits/participants, n	1056/422	1056/422	1127/450	421/1054	960/384
β (95%CI)	0.07 (-0.22, 0.35)	0.02 (-0.17, 0.20)	-0.25 (-1.10, 0.61)	-0.12 (-0.28, 0.03)	-0.21 (-0.71, 0.30)
VAS global					
Visits/participants, n	1093/441	1093/441	1165/469	440/1091	995/401
β (95%CI)	-0.04 (-0.31, 0.23)	0.09 (-0.09, 0.26)	-0.06 (-0.89, 0.77)	-0.08 (-0.23, 0.07)	-0.09 (-0.58, 0.39)
SJC-66					
Visits/participants, n	1245/492	1245/492	1321/521	491/1242	1136/448
β (95%CI)	-0.02 (-0.06, 0.02)	-0.008 (-0.03, 0.02)	0.01 (-0.10, 0.13)	-0.003 (-0.03, 0.02)	0.02 (-0.06, 0.09)
TJC-68					
Visits/participants, n	1245/492	1245/492	1321/521	491/1242	1136/448
β (95%CI)	-0.02 (-0.07, 0.04)	0.002 (-0.04, 0.04)	0.07 (-0.12, 0.26)	0.0005 (-0.04, 0.04)	-0.01 (-0.13, 0.10)
CRP					
Visits/participants, n	1245/492	1245/492	1321/521	425/1058	1136/448
β (95%CI)	-0.06 (-0.16, 0.04)	0.01 (-0.06, 0.08)	0.24 (-0.08, 0.56)	0.02 (-0.05, 0.08)	-0.07 (-0.26, 0.12)

Crude model: baseline PASDAS or DAPSA/component measure and study visit.
Adjusted model: baseline PASDAS or DAPSA/component measure, study visit, age, sex, education years, geographic origin, BMI, smoking, and alcohol consumption, and arthritis therapy.

Supplementary Table 7. Associations between meteorological factors on the average month before visit, and PASDAS and DAPSA and it components during the three years follow-up period

Disease activity	Temperature	Relative humidity	Wind speed	Atmospheric pressure	Sunshine duration
Crude model					
PASDAS					
Visits/participants, n	365/897	365/ 897	388/948	364/896	332/820
β (95%CI)	-0.01 (-0.03, 0.002)	0.05 (-0.01, 0.02)	0.01 (-0.06, 0.07)	-0.01 (-0.02, 0.01)	-0.02 (-0.05, 0.02)
DAPSA					
Visits/participants, n	901/371	901/371	954/395	370/899	825/339
β (95%CI)	-0.11 (-0.25, 0.006)	0.06 (-0.03, 0.14)	0.21 (-0.29, 0.71)	-0.08 (-0.19, 0.02)	-0.16 (-0.42, 0.09)
VAS pain					
Visits/participants, n	1056/422	1056/422	1127/450	421/1054	960/384
β (95%CI)	-0.04 (-0.37, 0.28)	0.01 (-0.22, 0.24)	-0.51 (-1.80, 0.77)	-0.25 (-0.51, 0.02)	-0.30 (-0.96, 0.36)
VAS global					
Visits/participants, n	1093/441	1093/441	1165/469	440/1091	995/401
β (95%CI)	-0.08 (-0.38, 0.23)	0.10 (-0.12, 0.31)	-0.37 (-1.57, 0.84)	-0.12 (-0.38, 0.13)	-0.09 (-0.71, 0.54)
SJC-66					
Visits/participants, n	1245/492	1245/492	1321/521	491/1242	1136/448
β (95%CI)	-0.03 (-0.07, 0.02)	-0.005 (-0.04, 0.03)	-0.03 (-0.21, 0.15)	0.03 (-0.02, 0.07)	-0.02 (-0.12, 0.08)
TJC-68					
Visits/participants, n	1245/492	1245/492	1321/592	491/1242	1136/521
β (95%CI)	-0.02 (-0.09, 0.05)	0.01 (-0.04, 0.06)	-0.04 (-0.32, 0.23)	0.03 (-0.03, 0.10)	-0.004 (-0.15, 0.4)
CRP					
Visits/participants, n	1061/426	1061/426	1117/451	425/1058	972/389
β (95%CI)	-0.07 (-0.16, 0.03)	0.02 (-0.06, 0.10)	**0.51 (0.11, 0.91)***	0.001 (-0.11, 0.11)	-0.12 (-0.33, 0.08)
Adjusted model					
PASDAS					
Visits/participants, n	365/897	365/897	388/948	364/896	332/820
β (95%CI)	-0.01 (-0.03, 0.003)	0.01 (-0.002, 0.02)	-0.01 (-0.08, 0.05)	-0.01 (-0.02, 0.01)	-0.02 (-0.05, 0.02)
DAPSA					
Visits/participants, n	901/371	901/371	954/395	370/899	825/339
β (95%CI)	-0.09 (-0.20, 0.03)	0.08 (-0.001, 0.17)	0.10 (-0.39, 0.59)	-0.09 (-0.19, 0.02	-0.18 (-0.43, 0.06)

VAS pain					
Visits/participants, n	1056/422	1056/422	1127/450	421/1054	960/384
β (95%CI)	0.08 (-0.24, 0.40)	0.02 (-0.20, 0.25)	-1.09 (-2.36, 0.19)	-0.25 (-0.52, 0.02)	-0.13 (-0.78, 0.52)
VAS global					
Visits/participants, n	1093/441	1093/441	1165/469	440/1091	995/401
β (95%CI)	-0.01 (-0.31, 0.29)	0.14 (-0.07, 0.36)	-0.71 (-1.90, 0.48)	-0.15 (-0.41, 0.11)	-0.06 (-0.67, 0.55)
SJC-66					
Visits/participants, n	1245/492	1245/492	1321/521	491/1242	1136/448
β (95%CI)	-0.02 (-0.06, 0.02)	-0.001 (-0.03, 0.03)	0.01 (-0.14, 0.17)	-0.002 (-0.04, 0.04)	-0.02 (-0.11, 0.07)
TJC-68					
Visits/participants, n	1245/492	1245/492	1321/592	491/1242	1136/521
β (95%CI)	-0.01 (-0.08, 0.05)	0.02 (-0.02, 0.07)	0.003 (-0.26, 0.26)	-0.004 (-0.07, 0.06)	-0.03 (-0.17, 0.11)
CRP					
Visits/participants, n	1061/426	1061/426	1117/451	425/1058	972/389
β (95%CI)	-0.08 (-0.19, 0.02)	0.04 (-0.04, 0.13)	**0.41 (0.01, 0.82)***	0.01 (-0.11, 0.12)	-0.19 (-0.42, 0.04)

Crude model: baseline PASDAS or DAPSA/component measure and study visit.
Adjusted model: baseline PASDAS or DAPSA/component measure, study visit, age, sex, education years, geographic origin, BMI, smoking, and alcohol consumption, and arthritis therapy.
* p-value<0.05

Supplementary Table 8. Associations between season and mood during the three years follow-up period

	Autumn	Winter	Spring	Summer

Crude model				
"I feel tense or wound up"				
Visits/participants, n	259 /118	224/101	173/81	233/102
β (95%CI)	**-0.14 (-0.28, -0.006)***	-0.12 (-0.27, 0.03)	-0.13 (-0.9, 0.03)	Ref
"I feel cheerful"				
Visits/participants, n	259/118	224/101	173/81	233/102
β (95%CI)	-0.02 (-0.14, 0.10)	-0.02 (-0.15 0.11)	-0.05 (-0.19, 0.09)	Ref
"I can sit at ease and feel relaxed"				
Visits/participants, n	259/118	224/101	173/81	233/102
β (95%CI)	0.11 (-0.03, 0.24)	0.11 (-0.04, 0.25)	0.10 (-0.06, 0.25)	Ref
Adjusted model				
"I feel tense or wound up"				
Visits/participants, n	259/118	224/101	173/81	233/102
β (95%CI)	-0.11 (-0.25, 0.02)	-0.09 (-0.24, 0.06)	-0.05 (-0.21, 0.10)	Ref
"I feel cheerful"				
Visits/participants, n	259/118	224/101	173/81	233/102
β (95%CI)	0.02 (-0.10, 0.14)	0.04 (-0.09, 0.17)	0.04 (-0.10, 0.18)	Ref
"I can sit at ease and feel relaxed"				
Visits/participants, n	259/118	224/101	173/81	233/102
β (95%CI)	0.10 (-0.04, 0.23)	0.12 (-0.03, 0.26)	0.08 (-0.07, 0.24)	Ref

Crude model: baseline mood measure and study visit.

Adjusted model: baseline component measure, study visit, age, sex, education years, geographic origin, BMI, smoking, and alcohol consumption, and arthritis therapy.

* p-value<0.05

Supplementary Table 9. Associations between season and psoriatic arthritis disease activity scores during the three years follow-up period by age groups

	Age<45				Age ≥45			
Disease activity	Autumn	Winter	Spring	Summer	Autumn	Winter	Spring	Summer
Crude model								
DAPSA								
Visits/participants, n	114/46	91/44	66/32	106/48	263/119	232/102	182/65	177/77
β (95%CI)	2.59 (-0.03, 5.21)	1.43 (-1.39, 4.26)	1.76 (-1.28, 4.79)	Ref	1.10 (-1.17, 3.37)	**2.95 (0.57, 23.34)***	**2.75 (0.29, 5.22)***	Ref
PASDAS								
Visits/participants, n	113/46	85/38	60/30	109/47	260/116	234/101	183/66	176/72
β (95%CI)	0.23 (-0.11, 0.56)	0.29 (-0.09, 0.67)	0.18 (-0.23, 0.59)	Ref	0.05 (-0.20, 0.29)	0.17 (-0.09, 0.43)	0.16 (-0.11, 0.42)	Ref
Adjusted model								
DAPSA								
Visits/participants, n	114/46	91/44	66/32	106/48	263/119	232/102	182/65	177/77
β (95%CI)	**3.20 (0.64, 5.75)***	0.46 (-2.34, 3.25)	1.31 (-1.65, 4.27)	Ref	1.28 (-0.98, 3.54)	**3.14 (0.79, 5.49)****	2.37 (-0.11, 4.85)	Ref
PASDAS								
Visits/participants, n	113/46	85/38	60/30	109/47	260/116	234/101	183/66	176/72
β (95%CI)	**0.35 (0.03, 0.68)***	0.25 (-0.12, 0.61)	0.12 (-0.26, 0.51)	Ref	0.06 (-0.18, 0.30)	0.14 (-0.11, 0.39)	0.10 (-0.16, 0.36)	Ref

DAPSA, Disease Activity index for Psoriatic Arthritis; PASDAS, Psoriatic Arthritis Disease Activity Score.
Crude model: baseline disease activity and study visit
Adjusted model: baseline disease activity, study visit, sex, education years, geographic origin, BMI, smoking, and alcohol consumption, and arthritis therapy.
** p-value<0.01. * p-value<0.05
